# Superb microvascular imaging technique in depicting vascularity in focal liver lesions: more hypervascular supply patterns were depicted in hepatocellular carcinoma

**DOI:** 10.1186/s40644-019-0277-6

**Published:** 2019-12-30

**Authors:** Fan Yang, Jing Zhao, Chunwei Liu, Yiran Mao, Jie Mu, Xi Wei, Jinyan Jia, Sheng Zhang, Xiaojie Xin, Jian Tan

**Affiliations:** 1 Department of Ultrasound diagnosis and treatment, Tianjin Medical University Cancer Institute and Hospital, National Clinical Research Center for Cancer; Key Laboratory of Cancer Prevention and Therapy, Tianjin; Tianjin’s Clinical Research Center for Cancer, Tianjin Medical University, Tianjin, 300060 China; 2 Department of Cardiology, Tianjin Chest Hospital, Tianjin, China; 3 Department of Pathology, Tianjin Medical University Cancer Institute and Hospital, National Clinical Research Center for Cancer; Key Laboratory of Cancer Prevention and Therapy, Tianjin; Tianjin’s Clinical Research Center for Cancer, Tianjin, 300060 China; 4 Department of Nuclear Medicine, Tianjin Medical University General Hospital, Tianjin, 300000 China

**Keywords:** Superb microvascular imaging, Color Doppler flow imaging, Hepatocellular carcinoma, Microvessel density, Vascular index

## Abstract

**Purpose:**

To investigate the capacity of Superb Microvascular Imaging (SMI) to detect microvascular details and to explore the different SMI features in various focal liver lesions (FLLs) and the correlation between SMI and microvessel density (MVD).

Method: Eighty-three liver lesions were enrolled in our study, including 35 hepatocellular carcinomas (HCCs) and 48 non-HCCs. All patients underwent color Doppler flow imaging (CDFI) and SMI examination and were categorized into subgroups according to Adler semiquantitative grading (grade 0–3) or the microvascular morphologic patterns (pattern a-f). The correlation between SMI blood flow signal percentage and MVD was assessed.

**Results:**

Compared with CDFI, SMI detected more high-level blood flow signals (grade 2–3) and more hypervascular supply patterns (pattern e-f) in HCCs (*p* < 0.05). Furthermore, more hypervascular supply patterns and fewer hypovascular supply patterns were detected in HCC compared with non-HCC (*p* < 0.05). Based on Adler’s grading or microvascular morphologic patterns, the areas under the receiver operating characteristic curve were 0.696 and 0.760 for SMI, 0.583 and 0.563 for CDFI. The modality of “SMI-microvascular morphologic pattern” showed the best diagnostic performance. There was significant correlation between MVD and the SMI blood flow signal percentage (vascular index, VI) in malignant lesions (*r* = 0.675, *p* < 0.05).

**Conclusion:**

SMI was superior to CDFI in detecting microvascular blood flow signals. More hypervascular supply patterns were depicted in HCC than in non-HCC, suggesting a promising diagnostic value for SMI in the differentiation between HCC and non-HCC. Meanwhile, we were the first to demonstrate that SMI blood flow signal percentage (VI) was correlated with MVD in malignant lesions.

## Background

Hepatocellular carcinoma (HCC) is the fourth most common cancer worldwide and one of the most common causes of cancer death [1]. Early detection and diagnosis are important for the prevention and treatment of HCC, and ultrasound (US) has been recommended as the first imaging modality for screening focal liver lesions (FLLs) because it is noninvasive and widely available. Basing on their characteristic appearance, common B-mode and color Doppler US can detect focal liver lesions, such as focal nodular hyperplasia (FNH) with a spoke-wheel shaped vascular pattern. The suggestive signs of malignancy are irregular walls or boundaries, hypervascularity with centripetal blood flow, basket appearance and high peak systolic flow velocity [2–4]. The characteristics of HCC, including its abundant blood supply and its easy metastasis and recurrence, suggest that angiogenesis plays an important role in tumorigenesis [5]. Color Doppler flow imaging (CDFI) is the most commonly used ultrasonic examination to detect blood flow. However, it is poorly effective in depicting slow blood flow and deep small vessels. For conventional US technique, it still remains a diagnostic challenge to identify the type of FLLs precisely and to distinguish between HCC and non-HCC, especially in the presence of chronic liver disease or cirrhosis.

Imaging information regarding vascularity pattern with contrast enhancement provides an important clue for the accurate differentiation of FLLs, such as contrast-enhanced CT or MRI and contrast-enhanced ultrasound (CEUS). However, several drawbacks limit their wide use. The cost of contrast agent is higher than that of traditional US, and the iodinated contrast agents may trigger or worsen renal failure in older patients and may induce hypersensitivity reactions.

Superb Microvascular Imaging (SMI) is an innovative vascular imaging technique that can detect microvascular flow at low velocity without contrast enhancement [6]. By using a powerful and intelligent algorithm, SMI removes tissue motion and extracts flow signals, and the vascular information is then displayed as a monochrome or color map of flow. HCC is a hypervascular tumor characterized by neovascularization and vascular invasion. The microvessel density (MVD) inside the tumor has been widely demonstrated as a reliable index to evaluate angiogenesis [7]. MVD is reported to be significantly correlated with both tumor size in patients undergoing HCC resection and early recurrence after liver resection [8]. Recently, several research studies have reported a better vascular depiction using SMI. The present study aims to investigate the capacity of SMI to detect microvascular details in FLLs and to explore the different SMI features between HCC and non-HCC. In addition, the correlation between SMI blood flow signal percentage and MVD in malignant lesions is assessed.

## Methods

This prospective study was performed from January 2017 to December 2017 at Tianjin Medical University Cancer Institute and Hospital, and the research protocol was approved by the ethics committee of the hospital and was performed in accordance with the ethical standards laid down in the 1964 Declaration of Helsinki. Signed informed consent was obtained from each patient before they participated in the study.

### Patients

Eighty-three patients (35 HCCs and 48 non-HCCs; age range: 33–78 years; mean age: 51 ± 19 years) with ultrasound-visible liver lesions were recruited. Patients were excluded if their condition was combined with severe organ failure or any previous treatment of the same lesions, such as chemotherapy. A total of 35 patients were combined with liver cirrhosis, while 28 patients were combined with fatty liver. A total of 49 patients had solitary lesions, and the remaining 34 patients had multiple lesions, while the largest single mass was included for the latter patients. Of all 83 liver lesions, 44 malignant lesions (35 hepatocellular carcinoma, 4 metastases, 3 intrahepatic cholangiocarcinoma and 2 hepatic lymphoma) and 6 benign lesions (2 hemangiomas, 3 focal nodular hyperplasia, and 1 adenoma) were pathologically diagnosed by surgery or needle biopsy, while 33 benign lesions (17 hemangiomas, 9 focal nodular hyperplasia and 7 adenoma) were radiologically confirmed based on typical contrast-enhanced CT and/or MRI imaging appearance. Differentiation of 35 HCCs included 20 moderately differentiated, 8 well-differentiated, and 7 poorly differentiated HCCs.

### Ultrasound examination technique

A Toshiba Aplio 500 US scanner (Toshiba Medical Systems, Tokyo, Japan) with a 1.5 to 6.0 MHz convex array probe was used to perform all US examinations. Patients were placed in the supine position with fully exposed abdomen after 6 h of fasting and were required to hold breath to minimize the negative effect of breathing. Motion artifact had a negative influence on SMI examination, so the FLLs adjacent to heart or abdominal aorta in left lobe were excluded. First, B-mode ultrasonography was thoroughly performed to scan the whole liver, and the general features of FLLs were observed (e.g., size, morphology, margin, and echo). When CDFI defined the section with most abundant blood supply, SMI was performed to depict the vascular structures of the FLLs. The following parameters were set for the SMI examination: color velocity scale 1 to 2 cm/s, color frequency 5–7 MHz, color frequency frame rate > 30 fps, and gain setting adjusted to show optimal imaging. Both color SMI (cSMI) and monochrome SMI (mSMI) were obtained in all subjects, but mSMI was used to assess the tumor vascularity in this study on account of its higher sensitivity. One patient received two consecutive examinations in one hour by two skilled radiologists with more than five years of clinical experience independently. The radiologists were blinded to contrast-enhanced CT and/or MRI imaging reports. When there was a discordance, US patterns were reviewed jointly to agree on a final diagnosis.

**Fig. 1:**
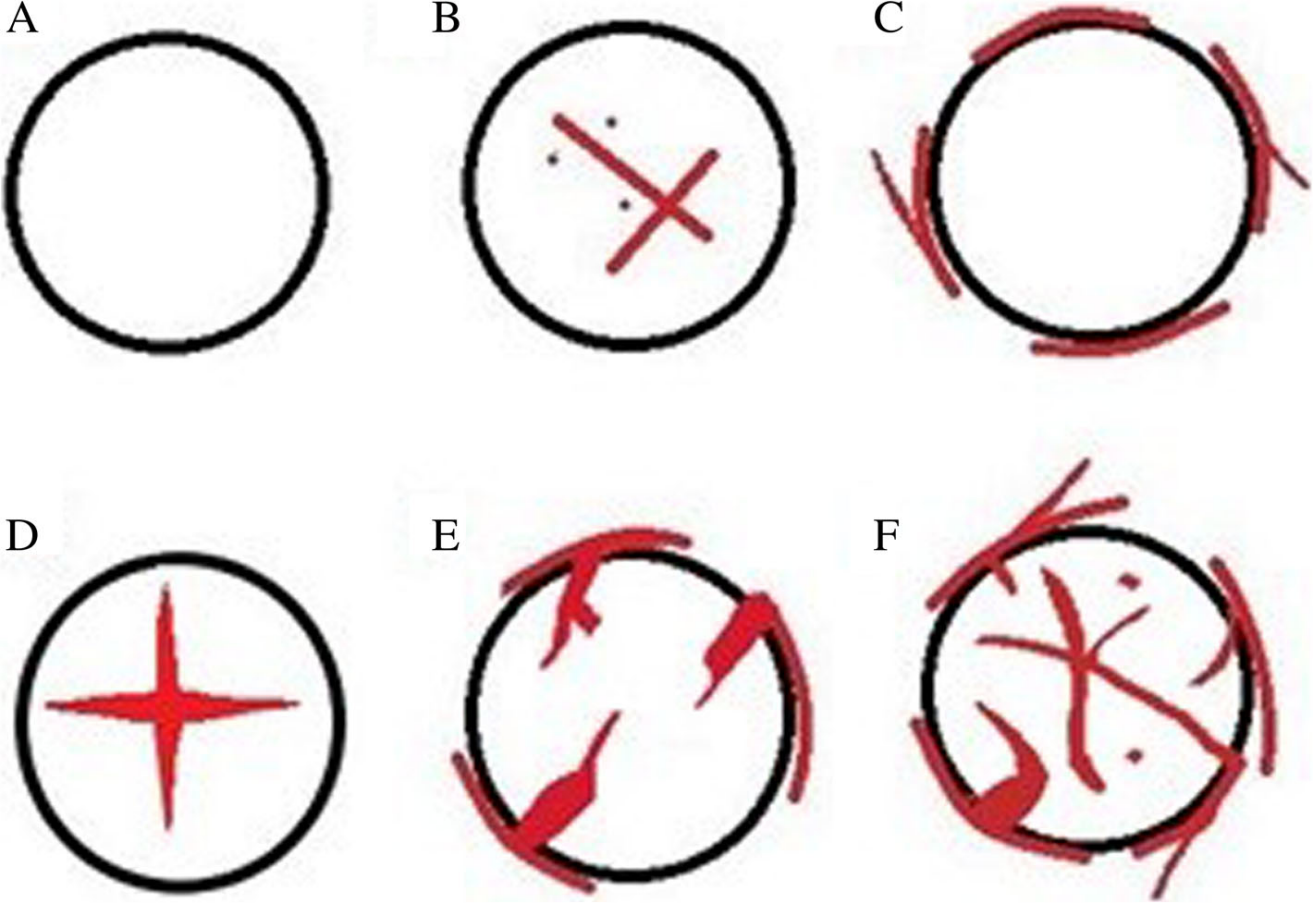
A simplified diagram of different morphologic patterns. **a**, no signal; **b**, dot-like or linear flow signal; **c**, nodular rim signal; **d**, spoked-wheel signal; **e**, residual-root or crab-claw flow signal: the dilated vessel was suddenly interrupted or divided into several slender vessels; **f**, irregular blood flow: vessels with irregularly tortuous course and different lumen diameter, often accompanied by large central feeding arteries

We used two different methods to categorize microvascular architecture patterns. Adler semiquantitative grading was used to classify blood flow detected within the tumor into four grades [9]: grade 0, no blood flow signals; grade 1, one or two dot-like or thin and short-like blood flow signals; grade 2, up to three dot-like blood flow signals or one longer blood flow signal; and grade 3, more than five dot-like blood flow signals or two longer blood flow signals. Morphologic features of vessels were divided into six patterns basing on previous reports [10, 11] (Fig. 1): a, no signal; b, dot-like or linear flow signal; c, nodular rim signal; d, spoked-wheel flow signal; e, residual-root or crab-claw flow signal; and f, irregular blood flow. A, b, c and d patterns were defined as hypovascular supply, while e and f patterns were defined as hypervascular supply.

The vascular index (VI) was used to estimate microvessel proportion on SMI, which was identified as the area percentage of blood flow in the focal lesion. After depicting the microvascular structures of the malignant lesions, the SMI images were stored, and the different pixel areas were calculated by ImagePro Plus software (Media Cybernetics, Inc., USA), VI = area of blood flow signal / area of region of interest.

### Immunohistochemistry and assessment of MVD

The streptavidin–biotin immunoperoxidase technique was used for immunohistochemical staining with monoclonal antibody CD34 (Antibody Diagnostic Inc., U.S.A.) on 4-mm paraffin sections as described [12]. A monoclonal anti-CD34 antibody was used at a 1:100 dilution at 4 °C for 12 h. The cystiform, dendritic, sinusoidal, or vacuolar structures contoured by endothelial cells that were stained yellow or brown by CD34 were considered as microvessels. Neither vessel lumen nor red blood cells were necessary for the definition of microvessels. The slide was viewed at low magnification (× 40–× 100) to find areas with the highest microvessel density. The stained blood vessels within five hot spots were then counted at high magnification (× 400). The minimum lumen diameter of the vessels revealed by CD34 ranged from approximately 10 to 40 μm. The final MVD was the average value obtained from the counts of the five fields.

### Statistical analysis

SPSS 19.0 software (IBM Corp., New York, NY) was used for all statistical analyses. The differences in blood flow signals and morphologic features of vessels between SMI and CDFI were analyzed by *X*^2^ tests or Fisher’s exact test. A receiver operating characteristic curve analysis was used to evaluate the diagnostic performance of SMI and CDFI. Kappa statistic was used to determine the interobserver agreement. The correlation between MVD and SMI was analyzed using Pearson’s test. *P* < 0.05 was considered statistically significant.

## Results

Among the 83 studied liver lesions, the mean diameter of HCC was 5.4 ± 2.4 cm, and the mean diameter of non-HCC was 3.1 ± 1.8 cm. There were no significant differences in SMI features between FLLs with different size (*p* > 0.05). 39 lesions located in the left lobe while 44 lesions located in the right lobe, and there were no differences assessing lesions between right lobe and left lobe (*p* > 0.05). The average depth was 46.8 ± 28.2 mm. The interobserver reproducibility was assessed by Kappa statistic. The κ-coefficients were 0.82 and 0.86 for Adler’s grading on CDFI and SMI and 0.84 and 0.80 for microvascular morphologic patterns on CDFI and SMI.

### Adler’s semiquantitative grading of vessels on CDFI and SMI

The results of Adler’s semiquantitative grading of all of the lesions are shown in Table 1. χ^2^ tests indicated that SMI was more sensitive in detecting blood flow in HCC compared with CDFI, meaning more high-level blood flow signals (grade 2–3) were revealed by SMI (*p* < 0.05). However, there were no significant difference between CDFI and SMI on non-HCC.

**Table 1:** Adler’s semiquantitative grading of vessels on CDFI and SMI, *p = 0.273, ^#^*p =* 0.033

The Adler’s semi-quantitative grading								
No.		hypovascular supply			hypervascular supply			
		Grade 0	Grade 1	combined	Grade 2	Grade 3	combined	*X*^2^
non-HCC 48	CDFI	18	24	42*	4	2	6*	1.2*
	SMI	8	30	38*	6	4	10*	
HCC 35	CDFI	8	16	24^#^	5	6	11^#^	4.546^#^
	SMI	3	7	10^#^	10	15	25^#^	

### Morphologic features of vessels on CDFI and SMI

**Fig. 2:**
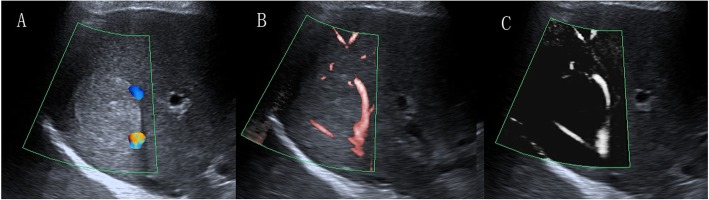
A 34-year-old man with hemangioma. **a** CDFI showed two vessel signals. **b** cSMI showed one long vessel signal (grade 2). **c** mSMI showed nodular rim signal (pattern D)

**Fig. 3:**
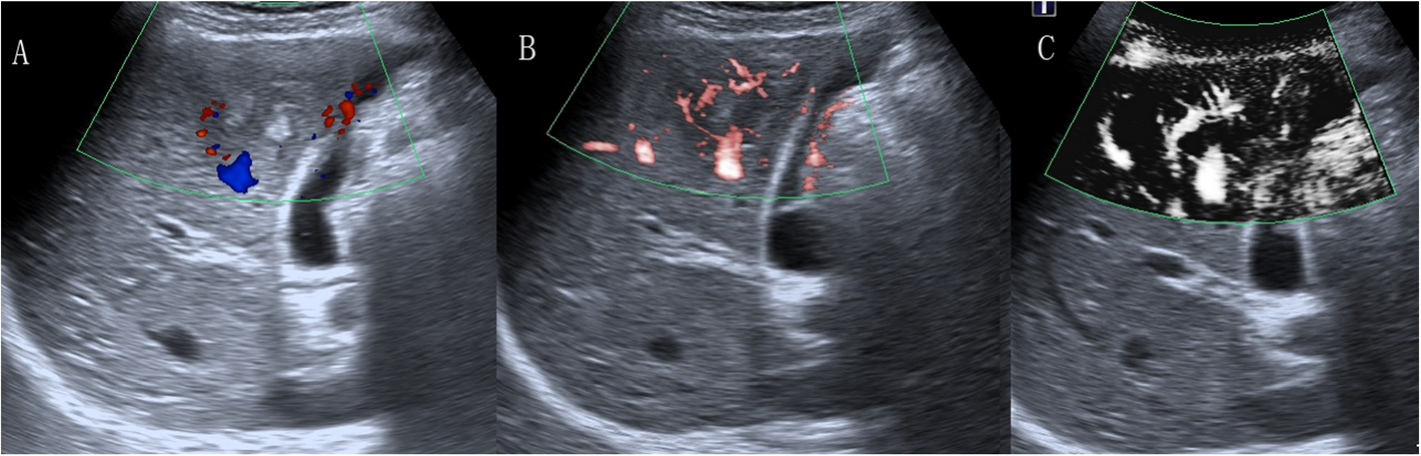
A 49-year-old woman with FNH. **a** CDFI showed two vessel signals (grade 1). **b** cSMI showed three long vessel signals (grade 3). **c** mSMI showed spoked-wheel signal (pattern D)

The morphologic features of vessels on SMI are summarized in Tables 2 and 3. The average size of the 19 hemangiomas was 2.4 ± 1.3 cm. Intrahepatic arterioportal shunts were found in 3 hemangiomas on enhanced MRI. The nodular rim pattern was the most common SMI feature of hemangioma (9/19, Fig. 2), followed by no signal pattern (4/19). The other hemangiomas exhibited dot-like or irregular flow signal patterns. Interestingly, the larger size of the hemangioma was prone to a concomitant increase in circular nodular rim signals. The average size of the 12 FNHs was 2.4 ± 0.9 cm. The SMI feature was characterized by a spoke-wheel pattern (5/12, Fig. 3), a dot-like or linear flow signal pattern (4/12) and no signal pattern (3/12). The mean size of adenomas was 5.3 ± 1.6 cm. SMI detected 3 nodular rim signal patterns (3/8), 3 irregular blood flow patterns (3/8) and 2 central dot-like or linear flow patterns (2/8). Concerning the malignant non-HCCs, the mean size of the 4 metastases was 3.1 ± 1.7 cm, with 2 dot-like or linear flow patterns and 2 no signal patterns observed on SMI. The mean size of the 3 intrahepatic cholangiocarcinomas was 2.3 ± 0.6 cm, with 2 intrahepatic cholangiocarcinomas exhibiting a dot-like or linear flow pattern and 1 intrahepatic cholangiocarcinoma exhibiting no signal pattern. The 2 hepatic lymphomas showed irregular vascularity on SMI. No significant difference was found in morphologic features between CDFI and SMI on these non-HCCs (*p* > 0.05).

**Table 2:** Morphologic features of vessels on SMI and CDFI, ^*^p = 0.537, ^#^*p =* 0.000

		*Morphologic features of vessels*								
		hypovascular supply (*N*)					Hypervascular supply (*N*)			*X*^*2*^
No.		a	b	c	d	combined	e	f	combined	
non-HCC	CDFI	18	17	7	1	43^*^	4	1	5^*^	0.381^*^
48	SMI	10	14	12	5	41^*^	0	7	7^*^	
HCC	CDFI	8	11	7	0	26^#^	4	5	9^#^	16.514^#^
35	SMI	3	2	4	0	9^#^	9	17	26^#^

**Table 3:** Different blood flow patterns between HCC and non-HCC on SMI, ^*^p < 0.05

	Morphologic pattern (*N*)							
	hypovascular supply					hypervascular supply		
*non-HCC*	*a*	*b*	*c*	*d*	combined	*e*	*f*	combined
Hemangiomas	4	4	9	–	17	–	2	2
Focal Nodular Hyperplasia	3	4	–	5	12	–	–	0
Adenoma	–	2	3	–	5	–	3	3
Metastases	2	2	–	–	4	–	–	0
Intrahepatic Cholangiocarcinoma	1	2	–	–	3	–	–	0
Hepatic lymphoma	–	–	–	–	0	–	2	2
*Total*					41^*^			7^*^
*HCC*	3	2	4	–	9	9	17	26
*Total*					9^*^			26^*^

**Fig. 4:**
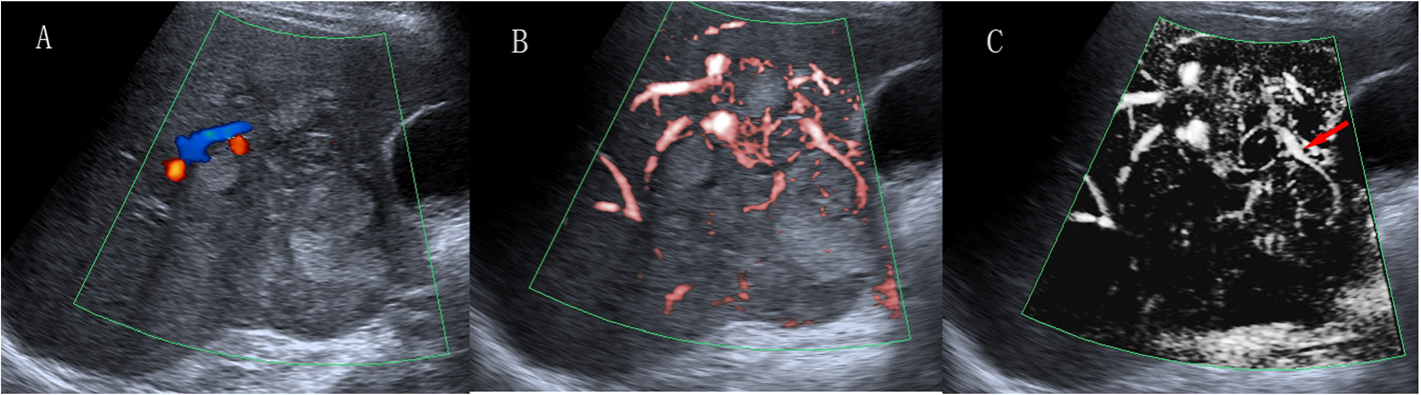
A 58-year-old woman with hepatocellular carcinoma. **a** CDFI showed three short-like blood flow signals (grade 2). **b** cSMI showed hypervascular supply in and surrounding the liver mass (grade 3). **c** mSMI showed residual-root flow signal pattern (the red arrow illustrates that the dilated vessel was divided into two slender vessels, pattern E)

**Fig. 5:**
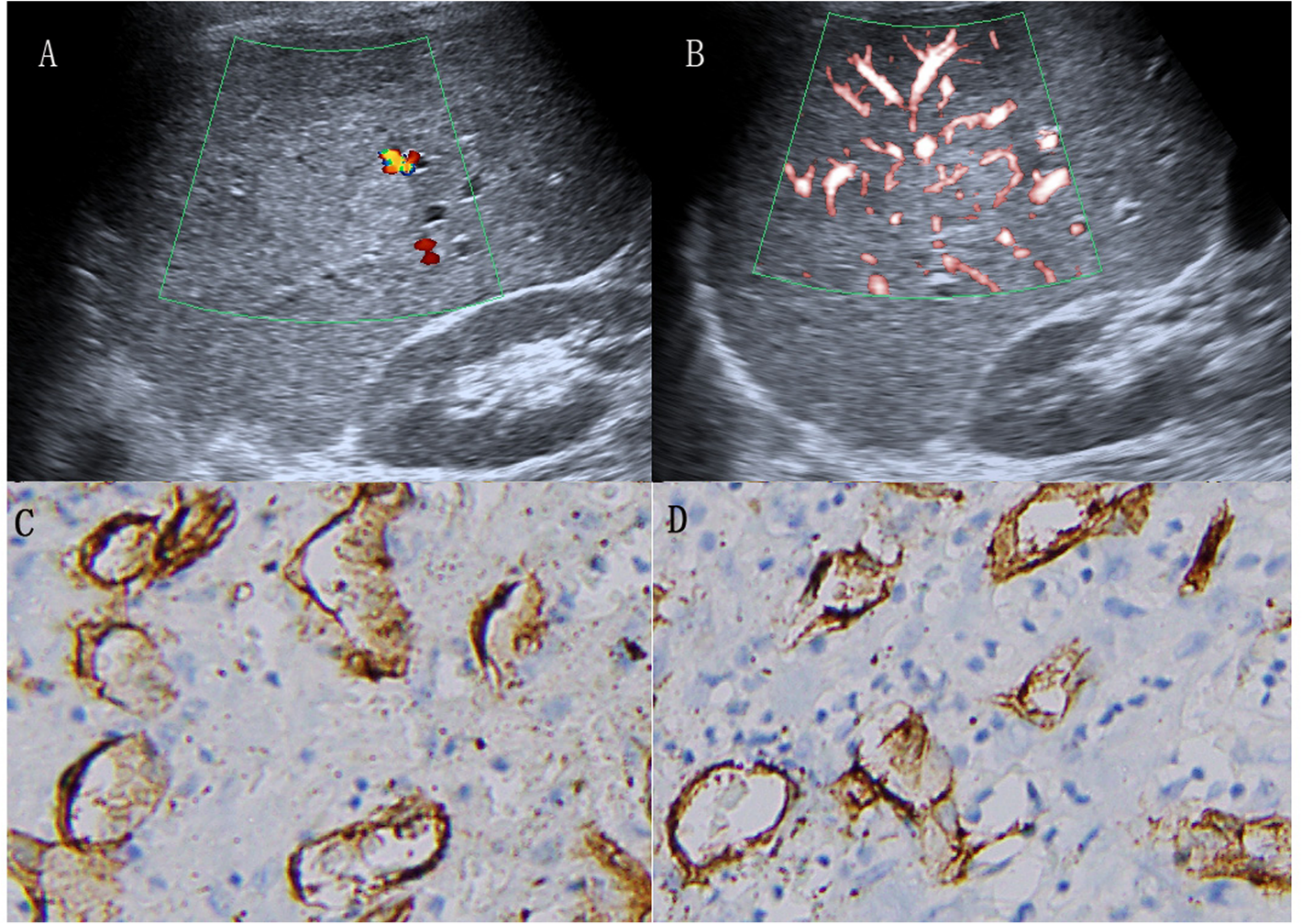
A 67-year-old man with hepatocellular carcinoma. **a** CDFI showed only one blood vessel signal (grade 1). **b** SMI showed abundant blood supply (grade 3) and irregular hypervascular supply (pattern F). **c** Immunohistochemical staining for CD34 indicated increased MVD in the center of the lesion. **d** Immunohistochemical staining for CD34 indicated increased MVD in the periphery of the lesion (original magnification, × 200)

**Fig. 6:**
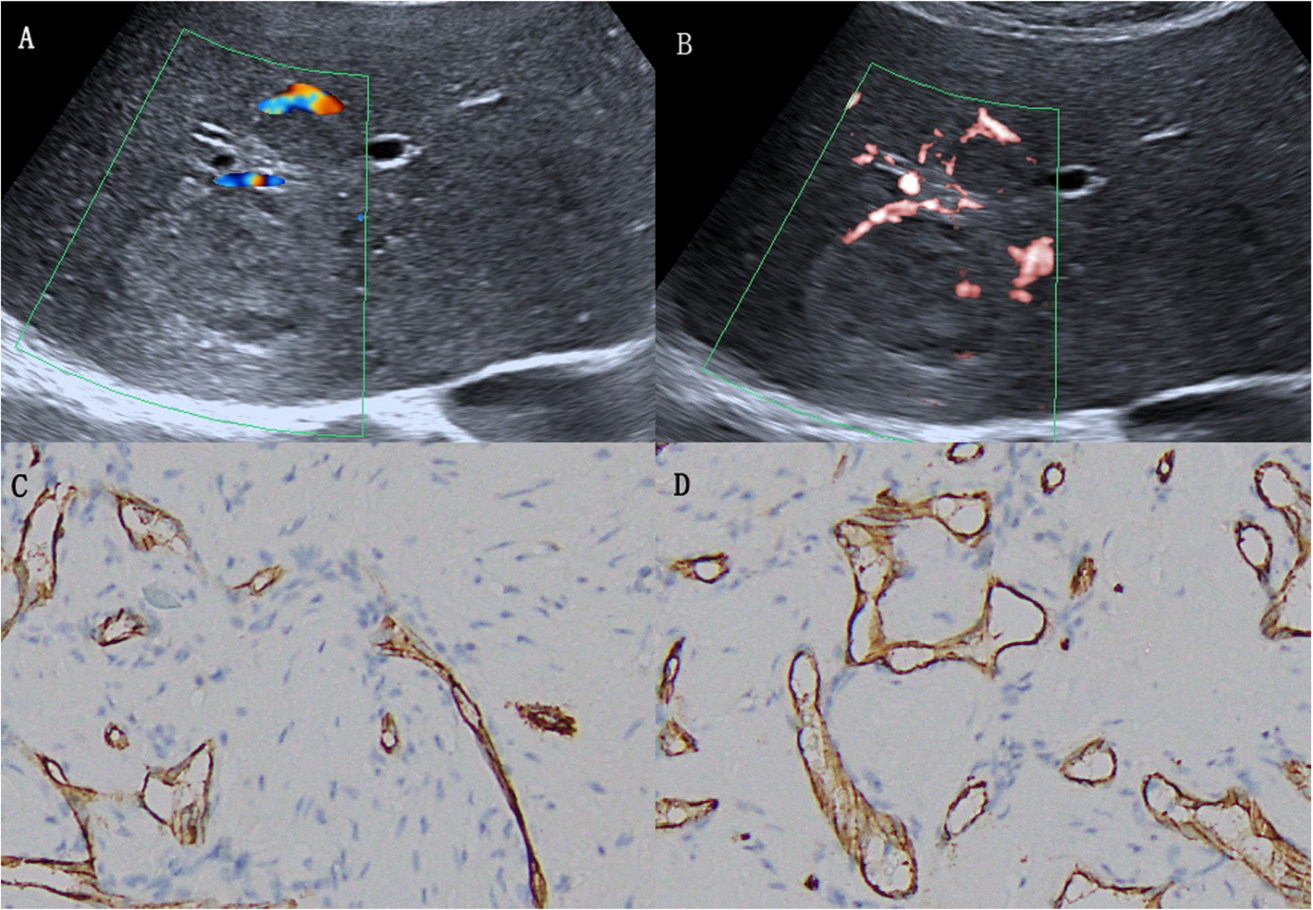
A 47-year-old man with hepatocellular carcinoma. **a** CDFI showed only one vessel signal (grade 1). **b** cSMI showed 3 vessel signals (grade 2) and nodular rim signal pattern (pattern C). **c** Immunohistochemical staining for CD34 indicated decreased MVD in the center of the lesion. **d** Immunohistochemical staining for CD34 indicated increased MVD in the periphery of the lesion (original magnification, × 200)

**Fig. 7:**
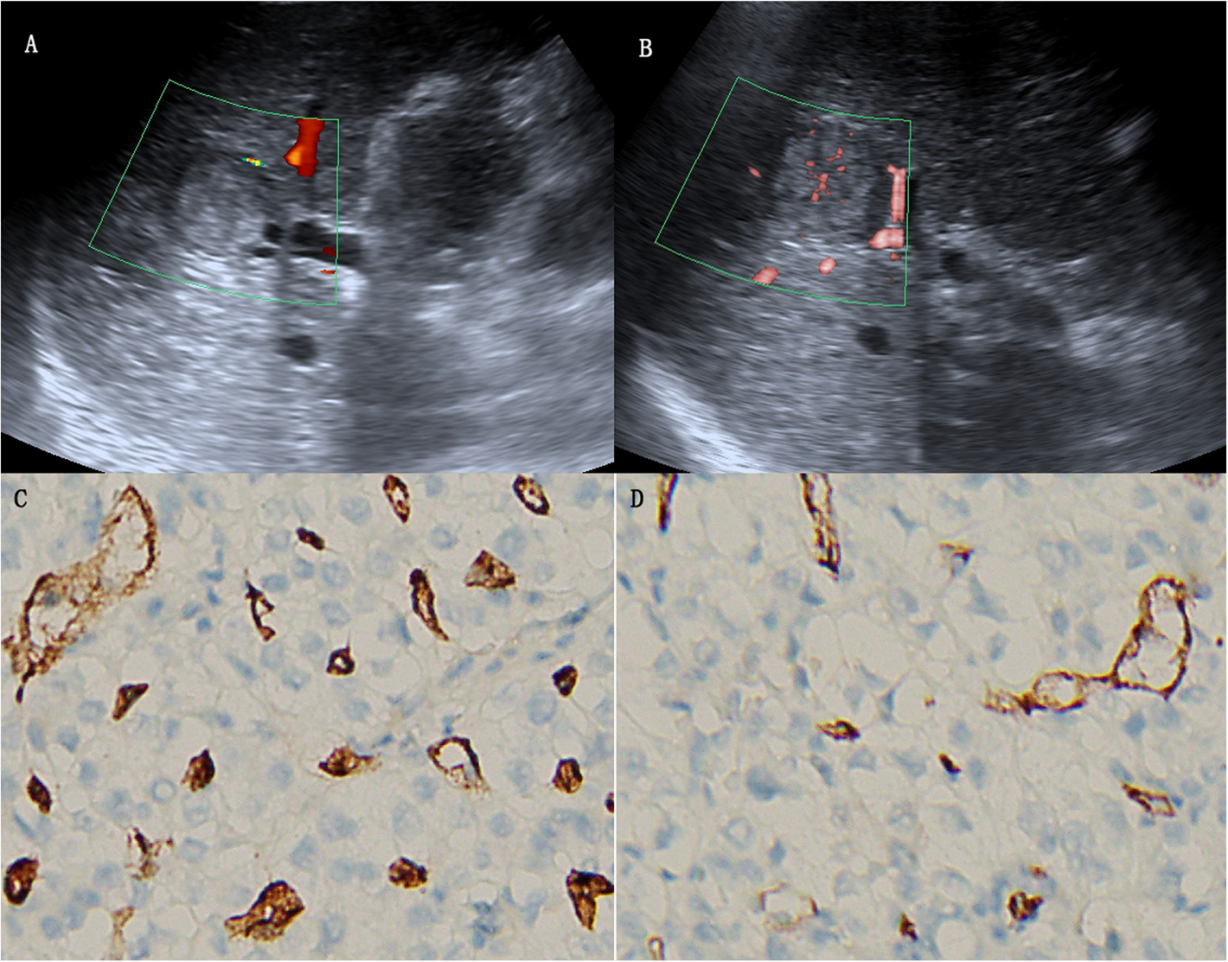
A 51-year-old woman with hepatocellular carcinoma. **a** CDFI showed 3 short vessel signals (grade 2). **b** cSMI showed more than five dot-like blood flow signals (grade 3) and irregular central feeding arteries (pattern F). **c** Immunohistochemical staining for CD34 indicated increased MVD in the center of the lesion. **d** Immunohistochemical staining for CD34 indicated decreased MVD in the periphery of the lesion (original magnification, × 200). CDFI = color Doppler flow imaging, cSMI = color Superb Microvascular Imaging, mSMI = monochrome Superb Microvascular Imaging. MVD = microvessel density

Regarding the 35 HCCs, SMI revealed 17 irregular blood flow patterns, 9 residual-root or crab-claw flow patterns, 4 nodular rim signal patterns, 3 no signal patterns and 2 dot-like patterns, with an average size of 5.4 ± 2.4 cm (Fig. 4, 5, 6 and 7).

Compared with CDFI, SMI revealed more hypervascular supply patterns (e and f patterns) and fewer hypovascular supply patterns (a, b, c and d patterns) in HCCs (Table 2, *p* < 0.05). Furthermore, SMI features were significantly different between HCC and non-HCC, meaning more hypervascular supply patterns (26/35 vs 7/48, *p* < 0.05) and fewer hypovascular supply patterns (9/35 vs 41/48, *p* < 0.05) were detected in HCC compared with non-HCC (Table 3).

### Diagnostic performance of CDFI and SMI

**Fig. 8:**
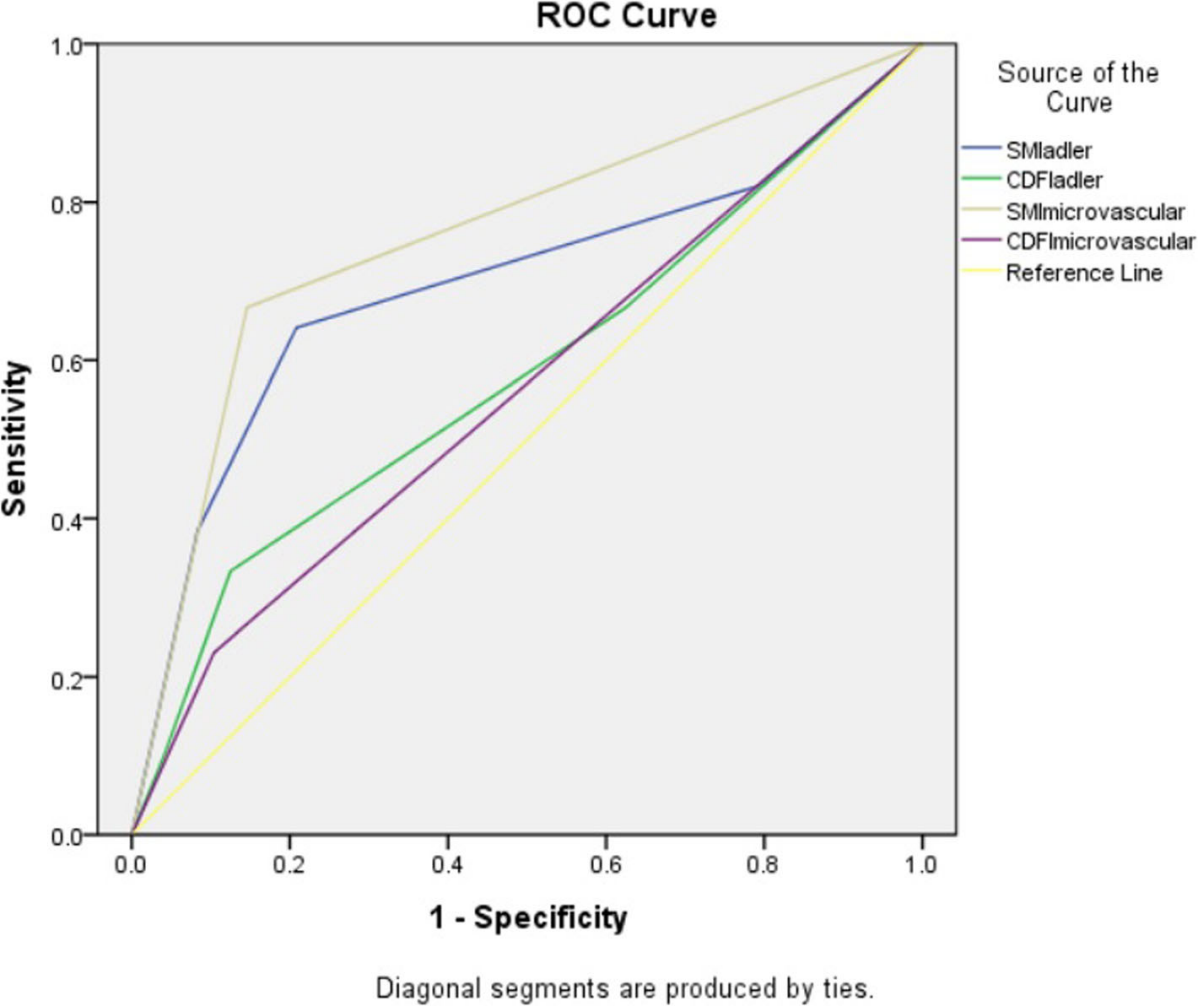
Performances of SMI and CDFI in the discrimination between HCC and non-HCC according to Adler’s grading and microvascular morphologic patterns, shown in a receiver operating characteristic curve

Using high-level blood flow signals (grade 2–3) and hypervascular supply patterns as diagnostic criteria for HCC, receiver-operating characteristic curves were generated to assess the diagnostic performance of CDFI and SMI (Fig. 8). The areas under the ROC curve (AUCs) for high-level blood flow signals on CDFI and SMI were 0.583 (31.4% sensitivity and 87.5% specificity) and 0.696 (71.4% sensitivity and 79.2% specificity), respectively, while AUCs for hypervascular supply patterns on CDFI and SMI were 0.563 (25.7% sensitivity and 89.6% specificity) and 0.760 (74.3% sensitivity and 85.4% specificity), respectively. The modality of “SMI-microvascular morphologic pattern” showed the best diagnostic performance among these modalities.

### Correlation between MVD and SMI in malignant lesions

**Fig. 9:**
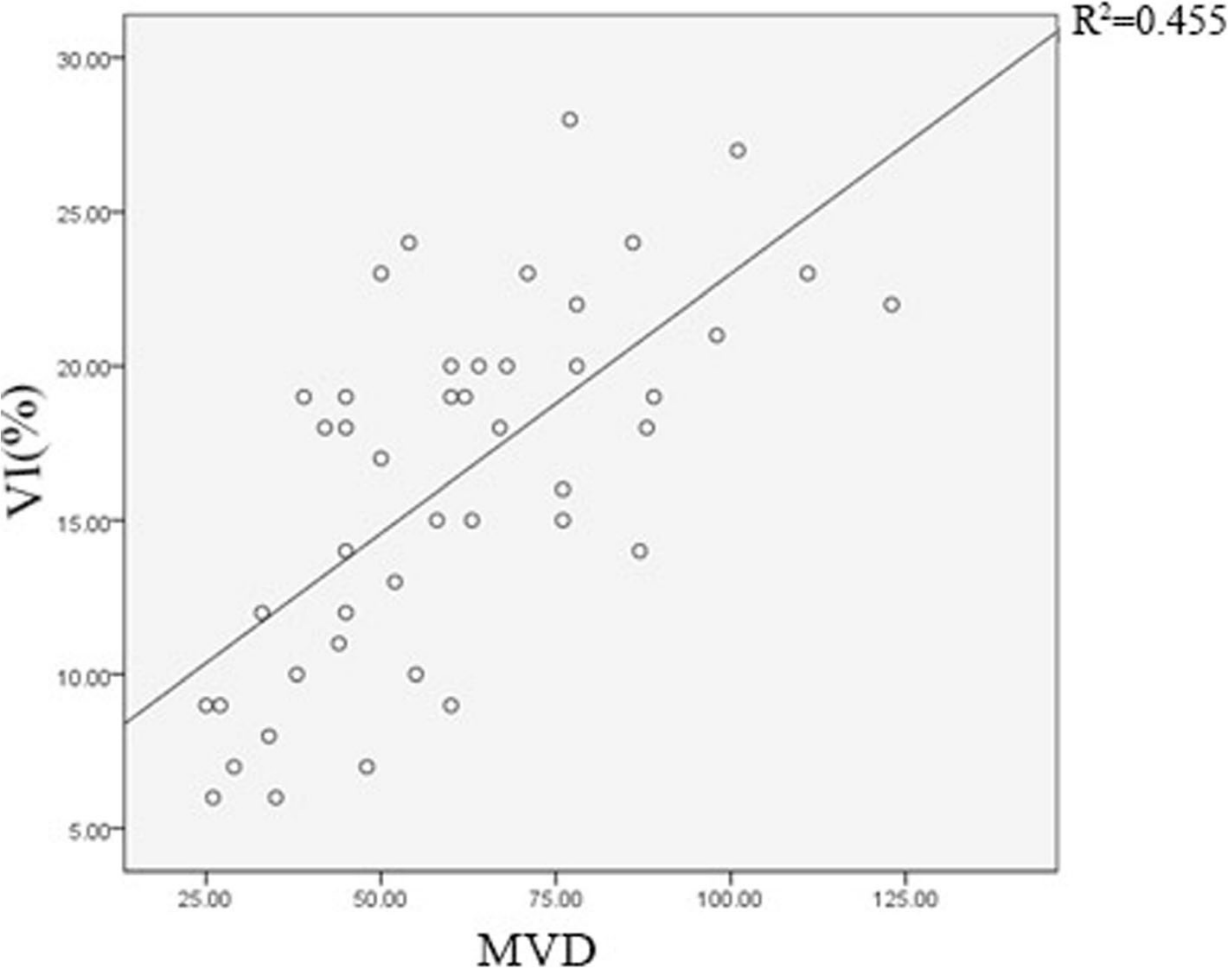
Scatter plot of MVD and VI. *r* = 0.675, *p* < 0.05

MVD examinations were performed in 44 malignant lesions after surgery, with an average value of 60.5 ± 23.5. The mean value of VI was 16.3 ± 5.9%. There was a significant correlation between MVD and VI (*r* = 0.675, *p* < 0.05, Fig. 9). Moreover, the abundant blood flow signal in local areas on SMI was prone to be accompanied with high MVD levels on postoperative pathological section. The correlation between SMI signal and MVD level in local area was illustrated in Fig. 5, 6 and 7.

## Discussion

As a novel technique, SMI has been demonstrated to be superior to CDFI in detecting microvascular details, owing to its sensitivity to low-velocity flow. The higher sensitivity and resolution recommend SMI as a valuable technique to evaluate microvascular structures without using any contrast agent [13]. Previous studies had demonstrated the feasibility of SMI in detecting ovarian vascularity in healthy children [14] or in distinguishing benign mass from malignant mass in renal tumor [15]. Park, et al. [16] reported that SMI revealed more vessels within 21 breast cancer patients than CDFI (the mean vessel number: 7.24 ± 3.0 vs 2.48 ± 2.4). Machado, et al. [17] compared the capacities of CDFI and SMI to evaluate lesions in thyroids and discovered that SMI had increased blood flow detection compared with CDFI and depicted more microvascular architecture details. Adler’s grading system has been used widely to assess vascularity based on the amount of blood flow visualized in a given area. Ma, et al. [6] reported that SMI was more sensitive in detecting higher-level blood flow (Adler’s grade 3) in malignant breast cancers. In this present study, we compared the capacities of CDFI and SMI to depict microvascular details in FLLs. Among the 35 HCCs, SMI identified 15 cases of Adler’s grade 3 level, while CDFI only detected 6 cases. Likewise, SMI revealed 26 cases of hypervascular supply patterns, while CDFI detected 9 cases of hypervascular supply patterns. Concerning the non-HCC, SMI and CDFI exhibited no statistical differences in the blood flow signal numbers and vessel morphology, which might be ascribed to the inadequate vascularization of these lesions.

Up to this point, few studies have focused on the different patterns of vessel morphology categorized by SMI features. Dong Ho Lee [10] reported that nodular rim patterns and spotty dot-like patterns were specific findings in hemangiomas, while spoke-wheel patterns and radiating vessel patterns were most commonly observed in FNHs. Our study demonstrated that more peripheral surrounding blood flow patterns were observed in hemangiomas. This was probably because the interface between hemangiomas and liver parenchyma is composed of thin-walled, dilated, blood-filled vascular sinus [18]. These findings are favored by the conception that hemangiomas exhibit peripheral nodular enhancement in the early arterial phase on contrast-enhanced ultrasound [19]. In spite of abundant vascular channels, the internal low-velocity venous blood flow was difficult to detect by CDFI but is easily detected by SMI, where it is presented as dot-like or linear flow signals.

The typical histologic features of FNHs consist of lobulated nodules separated by radiating fibrous tissue originating from a central scar [20]. The scar and radiating septum contain malformed vascular structures (such as tortuous arteries, capillaries, and veins), which presented as a central spoke-wheel pattern on SMI imaging in our study. This finding was consistent with a previous report by Bonacchi [21]. Though CDFI could detect the blood flow signals in approximately 30–40% of FNHs, SMI was more sensitive and accurate for detecting those small lesions.

Generally, tumor angiogenesis in HCC tends to show the following characteristics: increased vessel number, dilated vessel lumen, irregularly tortuous course and unintegrated basement membrane [22]. The above factors result in a significant increase in tumor blood perfusion. Previous studies with different modalities (contrast-enhanced CT/MRI [23], contrast-enhanced US [24]) have demonstrated this hypervascular appearance in HCC. Jang et al. [25] reported that hypervascularity was detected by CEUS in 87% of cases of HCC in the arterial phase, and dysmorphic arteries were seen in 72% of cases of HCC. He, et al. [11] showed that SMI patterns of HCCs and metastatic lesions were significantly different from those of hemangioma. Xu, et al. [26] used three-dimensional power Doppler imaging to depict vascularity in HCC, and their study suggested that hypervascularity with basket or internal vascularity patterns were the major patterns in HCC. The prevalence of a central feeding artery in HCC was also proved by arteriography and CT angiography [27]. In our study, SMI revealed 9 (25.7%) residual-root or crab claw-like flow patterns and 17 (48.6%) irregular flow patterns that were often accompanied by large central feeding arteries. These microvessel morphologic features reflected the vascular remodeling that originated from microvascular generation, suggesting a modest probability of HCC (AUC at 0.760, 74.3% sensitivity and 85.4% specificity). Using the peripheral or central vessel grades as diagnostic criteria to differentiate HCC from benign lesions, Theodore et al. [28] found a relatively low AUC value of 0.64. Consistent with our findings, Xiao, et al. [29] reported that root hair-like and crab claw-like patterns were specific to malignant breast lesions on SMI, with a higher AUC value of 0.88. In addition, SMI was also demonstrated significantly high sensitivity and accuracy for detection of intratumoral vascularity in suspected post-transarterial chemoembolization residual or recurrent HCC [30]. Further study is needed to evaluate the usefulness of SMI in the differential diagnosis between virous FLLs.

Intratumor microvessel density, a quantitative measurement of tumor angiogenesis, had been considered as a useful prognostic marker in many patterns of malignancy, including HCC [31]. Previous studies have explored the correlation between MVD and CDFI parameters and did not observe a significant correlation between MVD and peak flow velocity in either breast tumors [32] or uterine myomas [33]. The vascularity depicted by SMI was a combination of feeding artery, tumor vessels and draining veins. The minimum lumen diameter observed by pathology was much smaller than that of SMI by almost an order of magnitude (300 to 500 μm length in SMI); thus, SMI could not directly detect the microvessels observed by MVD. However, for the first time, our study demonstrated a significant correlation between MVD and SMI blood flow signal percentage regarding hypervascularity in HCC, using a more precise index: the vascular signal percentage (VI). Depicting the microvascular blood flow with very slow velocities, SMI provided more details associated with microvascular architecture that were previously sonographically undetectable. Quantitative analysis of VI is expected to provide more objective information which could be valuable for discriminating HCC from benign tumors. Meanwhile, MRI was used to measure tumor vessel permeability and tissue plasma volume for the functional assessment of neovascularization. The comparison between SMI and MRI in tumor angiogenesis is currently under investigation.

The present study had some limitations. First, this was a preliminary study performed at a single academic medical center with a relatively small sample size of patients. The sample sizes for lymphoma, intrahepatic cholangiocarcinoma and adenoma were simply too small to make definite inferences about imaging features. Therefore, a prospective clinical study with a larger scale is needed for an accurate portrait of different FLLs. SMI was a convenient and feasible technique and the time consumed on the evaluation was less than 15 min in most cases. However, a learning curve of 15–20 cases was needed for more proficient use of SMI in our experience. Second, CEUS was not evaluated in this study. In clinical practice, CEUS could depict the morphologic and distribution features of tumor microvessels, providing more diagnostic information. The combined diagnostic performance of SMI and CEUS may improve the diagnostic accuracy of HCC.

## Conclusions

In conclusion, SMI was superior to CDFI in detecting microvascular blood flow signals. More hypervascular supply patterns were depicted in HCC than in non-HCC, suggesting a promising diagnostic value for SMI in the differentiation between HCC and non-HCC. Meanwhile, we were the first to demonstrate that SMI blood flow signal percentage (vascular index) was correlated with MVD in malignant lesions.

## Data Availability

Not applicable.

## Abbreviations

SMI: Superb Microvascular Imaging
FLLs: focal liver lesions
MVD: microvessel density
HCCs: hepatocellular carcinoma
CDFI: color Doppler flow imaging
VI: vascular index
FNH: focal nodular hyperplasia
CEUS: contrast-enhanced ultrasound
